# Calculation of Configurational Entropy in Complex Landscapes

**DOI:** 10.3390/e20040298

**Published:** 2018-04-19

**Authors:** Samuel A Cushman

**Affiliations:** Rocky Mountain Research Station, US Forest Service, 2500 S Pine Knoll Dr., Flagstaff, AZ 86001, USA; scushman@fs.fed.us

**Keywords:** entropy, configuration, second law, Boltzmann entropy, Shannon entropy, landscape configuration, landscape pattern

## Abstract

Entropy and the second law of thermodynamics are fundamental concepts that underlie all natural processes and patterns. Recent research has shown how the entropy of a landscape mosaic can be calculated using the Boltzmann equation, with the entropy of a lattice mosaic equal to the logarithm of the number of ways a lattice with a given dimensionality and number of classes can be arranged to produce the same total amount of edge between cells of different classes. However, that work seemed to also suggest that the feasibility of applying this method to real landscapes was limited due to intractably large numbers of possible arrangements of raster cells in large landscapes. Here I extend that work by showing that: (1) the proportion of arrangements rather than the number with a given amount of edge length provides a means to calculate unbiased relative configurational entropy, obviating the need to compute all possible configurations of a landscape lattice; (2) the edge lengths of randomized landscape mosaics are normally distributed, following the central limit theorem; and (3) given this normal distribution it is possible to fit parametric probability density functions to estimate the expected proportion of randomized configurations that have any given edge length, enabling the calculation of configurational entropy on any landscape regardless of size or number of classes. I evaluate the boundary limits (4) for this normal approximation for small landscapes with a small proportion of a minority class and show it holds under all realistic landscape conditions. I further (5) demonstrate that this relationship holds for a sample of real landscapes that vary in size, patch richness, and evenness of area in each cover type, and (6) I show that the mean and standard deviation of the normally distributed edge lengths can be predicted nearly perfectly as a function of the size, patch richness and diversity of a landscape. Finally, (7) I show that the configurational entropy of a landscape is highly related to the dimensionality of the landscape, the number of cover classes, the evenness of landscape composition across classes, and landscape heterogeneity. These advances provide a means for researchers to directly estimate the frequency distribution of all possible macrostates of any observed landscape, and then directly calculate the relative configurational entropy of the observed macrostate, and to understand the ecological meaning of different amounts of configurational entropy. These advances enable scientists to take configurational entropy from a concept to an applied tool to measure and compare the disorder of real landscapes with an objective and unbiased measure based on entropy and the second law.

## 1. Introduction

The second law of thermodynamics is the governing principle of nature, and entropy is the central concept of the second law. Strangely, however, entropy and the second law are nearly absent from the landscape ecology literature [1,2]. The focus of landscape ecology is on understanding pattern-process relationships across scales in space and time [3]. Cushman [2] argued that since every interaction between entities leads to irreversible increases of entropy and decreases of free energy, it should follow that landscape patterns, processes of landscape change, and the propagation of pattern-process relationships across space are constrained and directed by the second law, and that entropy should be the operative measure to quantify, compare and predict landscape pattern-process relationships.

The fundamental connections between thermodynamics and entropy were noted in seminal works in the field of landscape ecology (e.g., [4,5,6,7], and in the subsequent decades a few researchers continued to develop thermodynamic ideas in landscape ecology (e.g., [8,9,10]. However, a recent review of the usage and application of the entropy concept in landscape ecology [1] unequivocally showed that landscape ecology has largely ignored thermodynamic theory, concepts or methods. This disjunction is the primary motivation for this special issue on the linkage between entropy and landscape ecology, and the purpose of this paper is to advance a recent line of work that formalizes the entropy concept in landscapes [2], and develops the theory of how to measure it [11,12]. The primary objective of this paper is to provide a practical analytical approach to measure and compare the entropies of real landscapes.

I proposed that the Boltzmann relation, *s* = *k*ln*W*, provides both a theoretical foundation and a computational solution for calculating the configurational entropy of landscapes [11]. The Boltzmann relation defines the entropy of a system as proportional to the logarithm of the number of microstates in the macrostate of that system, where the macrostate of the system is some broad-scale, emergent property, and the microstates are unique arrangements of the system that produce that macrostate. I suggest that this concept could be applied to landscapes by defining the macrostate as the amount of edge length between pixels of different cover classes in a landscape mosaic, and the microstate as the unique arrangements that the landscape mosaic could take (e.g., how many unique ways the pixels of that map can be arranged), and the entropy of the landscape is proportional to the logarithm of the number of arrangements of the lattice (microstates) that produce the same amount of total edge length (macrostate). Using this principle I show how to calculate the Boltzmann entropy of several simple landscape mosaics, and demonstrate that this configurational entropy measures the disorder of the landscape, such that both landscapes that are highly aggregated and those that are highly dispersed have low entropy, and random arrangements produce configurations that have an edge length with many microstates corresponding to high configurational entropy.

The utility of the application of Boltzmann entropy to real landscapes appears to be limited (e.g., [11,12]) given that it seems to depend on computing all the microstates of a landscape (e.g., all unique configurations), calculating the edge length of each and then calculating the number of arrangements that produce an edge length that matches that of the real, observed landscape. In landscapes of realistic dimensionality there are an intractably large number of possible arrangements, making it impossible to formally calculate *W*, the number of microstates in the macrostate [11]. There are several possible solutions to this difficulty. In [11] I proposed that the effectively infinite number of unique configurations in landscapes of realistic dimensionality means that one can use neutral models such as QRULE [13] to generate a large sample of spatially random maps, which can then be used to estimate the l number of microstates in a given macrostate, and the distribution of microstates in all possible macrostates of a landscape mosaic. This paper directly continues this line of thinking to demonstrate a practical solution calculating the relative Boltzmann configurational entropy for landscapes of any dimensionality.

The solution proposed in this paper is based on four key ideas. First, a distribution of microstates for any landscape mosaic can be computed by randomly permuting the lattice to produce random configurations of the same dimensionality. Second, following the central limit theorem, the total edge length in this distribution of randomized landscapes will follow a normal distribution. Third, by fitting a normal probability density function to this distribution of total edge length in the randomized microstates, we can predict the proportion of microstates with any given edge length. Fourth, taking an analogy from Shannon entropy [14], we can use the proportion of microstates (*p*), rather than the number proper (*W*), to compute a relative Boltzmann entropy that we can use to compare the relative entropy of landscapes with the same number of pixels and cover types. The goal of this paper is to evaluate each of these four assertions, demonstrate that they hold for a sample of real landscapes of varying dimensionality, patch richness and evenness of cover type proportionality, and finally provide equations that allow researchers to calculate the normal distribution of microstates associated with any landscape directly to enable rapid calculation of landscape entropy without the need to conduct onerous randomization analyses. 

## 2. Methods

**Figure 1:**
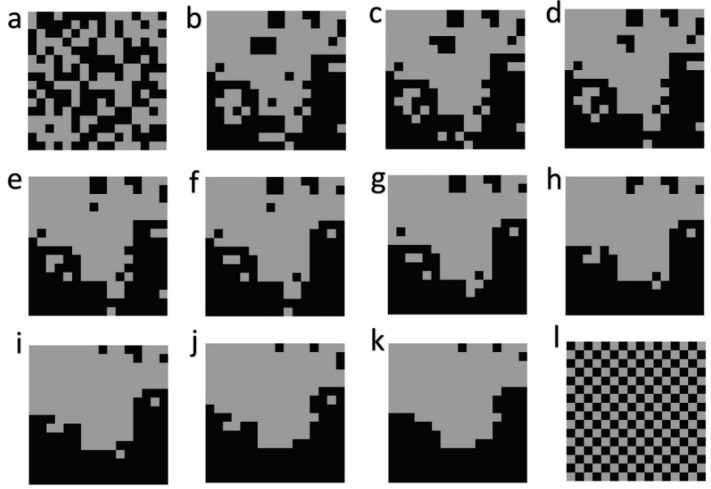
The 12 test landscapes. (**a**) random; (**b**) H1; (**c**) H2; (**d**) H3; (**e**) H4; (**f**) H5; (**g**) H6; (**h**) H7; (**i**) H8; (**j**) H9; (**k**) H10; (**l**) checker board.

The first component of this evaluation uses neutral models to produce 12 different landscape configurations for a landscape of 256 cells in 16 × 16 dimensionality, with two cover types, each with 50% area. I used QRULE [13] to produce a simple random map (Figure 1a) and multi-fractal maps with *H* parameter (which controls the degree of aggregation), ranging from 0.1 (highly fragmented) to 1 (highly aggregated), at 0.1 an interval of 0.1 H unit (Figure 1b–k), with a constant random number seed. Finally, to produce a highly dispersed pattern, I generated a perfect checkerboard (Figure 1l).

I produced a distribution of microstates for these landscapes by randomly permuting them. Simply permuting rows and columns does not fully randomize a landscape mosaic. To create a truly random distribution of configurations for these test landscapes I used a three-step process. First, I vectorized the maps into a single column of 256 rows. Second, I randomly permuted this vector. Third, I reordered the randomized vector into 16 × 16 lattices. For each of the 12 test landscapes I computed 100,000 random permutations (microstates).

I used FRAGSTATS (FRAGSTATS v4: Spatial Pattern Analysis Program for Categorical and Continuous Maps. Computer software program produced by the authors at the University of Massachusetts, Amherst. http://www.umass.edu/landeco/research/fragstats/fragstats.html) [15] to calculate the landscape metric total edge length on each of the 12 test landscapes and the 100,000 microstates for each. Total edge length computes the length (in meters) between pixels of different classes in a categorical landscape mosaic, and is the state variable to define the macrostate for landscape entropy [11]. To test the assertion that the distribution of the total edge length in the distribution of permuted landscapes would follow a normal distribution, I computed the histograms of total edge length for the 100,000 permutations of each test landscape, calculated the mean and standard deviation of these, and overlaid the normal distribution with that mean and standard deviation to verify that the permuted distribution followed the parametric probability function perfectly.

The normal probability function provides predictions for the frequency at which each value in the distribution will occur. I used this concept to compute the expected frequency of the observed total edge length in the 12 test landscapes among the full distribution of microstates for that test landscape.

To demonstrate the utility of the proportion of microstates rather than actual number in a macrostate to compute configurational entropy, I produced scatter plots of the relationship between Boltzmann entropy based on computing all actual microstates (ln*W*) and the logarithm of the probability of a given total edge length in the distribution of microstates for each test landscape (ln*p*). I used regression to quantify how closely lnp is related to the true Boltzmann entropy (ln*W*). Finally, using the relationship between ln*p* and ln*W*, I calculated the relative configurational entropy of each of the 12 test landscapes and compared them to each other to quantify the degree of disorder in each configuration.

In the second part of this evaluation I explored the boundary limits of the normal approximation for the distribution of total edge length of permuted landscape mosaics. The normal approximation is likely to break down in cases where landscapes are very small, defined by the number of rows and columns, and is very highly dominated by a majority class. This is because when there are relatively few pixels in a landscape and almost all of the pixels are a single class there are few different states possible for total edge length, and the permuted distribution of the total edge length will be truncated at the upper end due to the fact that the total edge length reaches a theoretical maximum when all minority pixels are singletons in isolation surrounded by majority pixels. I explored the boundary limits of the normal approximation by evaluating the normality of the distribution of the permuted total edge lengths for a 5 × 5 factorial (Table 1) consisting of five levels of landscape extent (20 × 20, 30 × 30, 40 × 40, 50 × 50 and 60 × 60) and five levels of minority proportionality in a two-class landscape (2%, 4%, 6%, 8%, 10%). Landscapes with larger size, a higher proportion of minority class or a larger number of classes will achieve normality more readily than the factors explored here. Therefore, this evaluation provides a strict and conservative evaluation of the boundary limits for the normal approximation.

**Figure 2:**
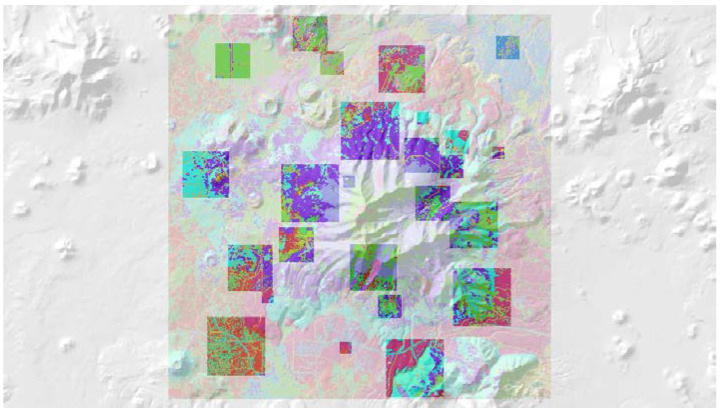
Location and pattern of the 25 sample landscapes in the San Francisco Peaks region of Northern Arizona. The extent of the 20 × 20 sample landscape is shown in semitransparent background overlaid on a hillshade of topography. The individual sample landscapes are shown with random colormap corresponding to each class in the cover type-seral stage classification.

In the third component of this evaluation I evaluated this method using a sample of 25 real landscapes of varying dimensionality (number of columns and rows), patch richness (number of different cover types) and evenness of the proportional amount of each cover type. Specifically, I evaluated a selection of sub-landscapes within a 20 × 20 km landscape centered on the San Francisco Peaks region of Northern Arizona, USA (Figure 2). I selected a landcover layer that represents vegetation cover type across seral stages, produced by the LANDFIRE program [16]. The layer has a 30 m spatial grain (pixel size), and consists of 50 total classes. I randomly selected five replicate landscapes, each of five extents, consisting of 20 × 20 (600 m), 40 × 40 (1200 m), 60 × 60 (1800 m), 80 × 80 (2400 m) and 100 × 100 (3000 m) extents. The lower left corners of these sub-landscapes were spatially randomly located, with the constraint that test landscapes were not allowed to overlap. For each test landscape I followed the same procedure used above in evaluating the QRULE landscapes. Specifically, I vectorized the landscapes and randomized them 100,000 times, calculated the total edge length on each randomized permutation with FRAGSTATS, calculated the mean and standard deviation of this randomized distribution, produced a frequency distribution of the observed frequencies of total edge length for the randomized distribution, and overlaid the associated normal distribution with the same observed mean and standard deviation to confirm the fit between the parametric normal distribution and the observed frequency distribution. Then I calculated the relative Boltzmann entropy for each replicate landscape using the parametric normal probability density function to estimate the number of microstates out of 10^100^ random permutations with the same total edge length, and plotted this on the theoretical distribution of macrostates for a landscape with that dimensionality, number of patch types and proportion of each patch type. Finally, I fit linear models to predict the mean and standard deviation of the distributions of macrostates (total edge length) of a landscape as a function of the dimensionality of the landscape, and the Fragstats metrics patch richness (number of cover types) and SHDI (the Shannon diversity of the landscape; [14]).

## 3. Results

### 3.1. Comparing Fully Permuted and Row–Column Permuted Distributions of Total Edge Length

I asserted that randomly permuting the rows and columns of a landscape mosaic does not fully randomize it, and it is necessary to fully randomize the lattice to produce an unbiased distribution of microstates. Comparison of the distributions of total edge length from a row-column permuted distribution and a fully vector-randomized distribution shows that this is the case. Specifically, the mean and standard deviation of the row-column permuted distribution were 200.0 and 13.4, respectively, while those of the fully vector-randomized distribution were 246.70 and 10.0, respectively. This shows that the row-column permutation does not remove all structure, since there is on average significantly (*t*-test *p* < 0.001) lower total edge length in the row–column permuted distribution than the vector-randomized distribution.

### 3.2. The Vector-Randomized Distribution of Total Edge Length Is Normally Distributed

**Figure 3:**
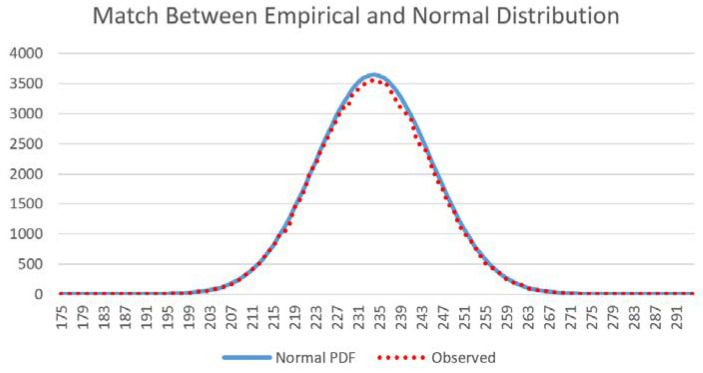
Match between the vector-permuted distribution of total edge length and the predictions of the normal probability function.

There was an exceptionally close match between the observed frequency of total edge length in the distribution of permuted landscape mosaics and the predictions of the normal probability density function with the same mean and standard deviation (Figure 3). In addition, each of the 12 test landscapes, though having very different configurational patterns (Figure 1), produce identical normally distributed microstates. Specifically, the normal distributions of total edge length for the vector-randomized test landscapes were distributionally identical, with the same means and standard deviations (*p* > 0.99999 for differences), and with histograms that were completely congruent. This shows that the distribution of microstates is independent of the macrostate, but only a function of the dimensionality and the number of classes in the landscape.

### 3.3. Relationship between lnW, pln(p) and lnp

**Figure 4:**
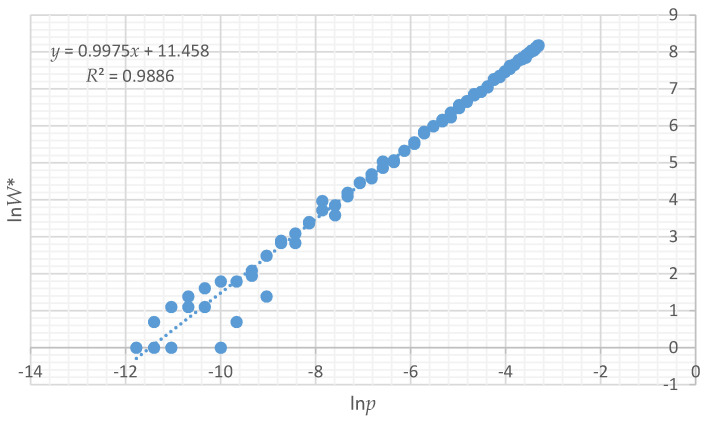
Relationship between configurational Boltzmann entropy (ln*W*) calculated from the vector-permuted distribution of all 12 test landscapes.

There is a very strong relationship between lnW* (logarithm of the number of instances of a given total edge length out of 100,000 permutations of the 16 × 16 landscape lattice), and ln*p* (relative Boltzmann entropy) (Figure 4). Specifically, lnW* and ln*p* are linearly related, with a slope of 1, and an intercept of 11.47 (Figure 4, R-squared 0.989), a very tight 1:1 linear relationship between the formal Boltzmann entropy lnW* and the relativistic form of Boltzmann entropy ln*p*.

**Figure 5:**
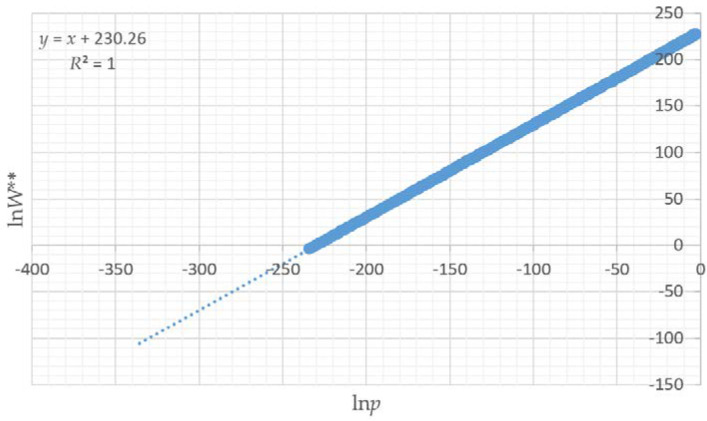
Verification that the linear relationship with slope 1 is consistent for a large number (10 × 10^100^) of computed configurations. W** is the number of microstates with a given total edge length expected from 10 × 10^100^ permutations of the lattice, using the normal probability density function.

Given the intractably large number of possible configurations that a lattice of 256 cells with 50% area in each of two classes can take (256!/(128! × 128!; [11]), it is not possible to compute the exact number of true microstates. Given that we cannot fit the canonical forms of the relationships between ln*p* and ln*W*, we therefore cannot estimate the true Boltzmann entropy (ln*W*) for each observed level of total edge length expressed in the 12 test landscapes. However, we can be confident that the linear and logarithmic relationships between ln*p* and ln*W* will be consistent. I verified this by computing the expected number of microstates (W**) with each amount of total edge length among 10 × 10^100^ permutations of the 256 cell lattice, using the parametric normal probability density function, and confirmed that the slope is constant at 1, and only the intercept changes (Figure 5). Therefore, the linear relationship between ln*p* and ln*W* gives us a means to compute the relative Boltzmann entropy for each test landscape. Specifically, the ratios of the ln*p* values for the different test landscapes provide a measure of the relative difference in the Boltzmann configurational entropies.

### 3.4. Configurational Entropy of the 12 Test Landscapes

Relying on the linear relationship with slope 1 between ln*p* and ln*W*, I computed the relative differences in the entropies of the 12 test landscapes (Table 2). This table shows the expected pattern of relative difference in entropy among the test landscapes, namely that the random configuration has by far the highest entropy, and that the perfect checkerboard has the lowest, with the entropy of the fractal landscapes decreasing with the increasing *H* level (increasing aggregation). The calculation quantifies these relative differences. For example, the random landscape (R) has an entropy 11.07 times greater than that of the patterned map that is nearest to it in entropy (H1), and has 58.82 times more entropy than the checkerboard map. The different fractal maps (H1–H10) also differ dramatically among each other in terms of relative configurational entropy. For example, the second most aggregated (H9) fractal map has 1.10 times more entropy than the most aggregated fractal map (H10) and the least aggregated fractal map (H1) has 3.55 times higher entropy than the most aggregated fractal map (H10). The pattern of relative entropies in Table 3 reveals several things. First, as expected, the random map has the highest relative entropy. Second, the entropy of the least aggregated fractal map is closer to that of the random map than that of the most aggregated fractal map. Third, the difference in entropy between the least aggregated fractal map and the random map is less than the difference in entropy between the most aggregated fractal map and the checkerboard configuration. These relative differences in entropy describe the differences among the test landscapes in their degrees of disorder, and provide an objective and quantitative measure of the degree of organization in each landscape configuration.

### 3.5. Shape of the Entropy–Total Edge Length Curve and Position of the Test Landscapes Along It

**Figure 6:**
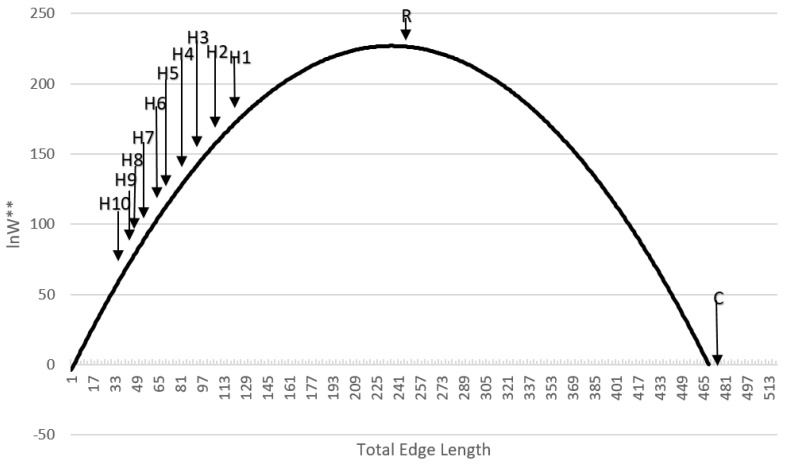
Location of the 12 test landscapes along the parabolic curve of entropy as a function of total edge length in the landscape.

Plotting the lnW** values against total edge length across the full range of values in the normal probability distribution function shows that the shape of the entropy-edge length relationship is parabolic (Figure 6). This follows from the mathematical facts that a log transformation of a Gaussian curve is a parabola, the frequency of total edge length in the permuted distribution of landscape lattices is normally distributed, and entropy is defined as the logarithm of the frequency of microstates (unique configurations) in a macrostate (amount of total edge length).

The locations of the test landscapes along this curve is instructive. First, the random landscape is located very near the center of the distribution, with nearly maximum possible entropy. Second, the aggregated multifractal maps are distributed to the left with relatively low total edge length relative to the permuted distribution, with entropy increasing with decreasing aggregation. Third, the checkerboard pattern is located far to the right at the extreme edge of the distribution. In fact, it is slightly beyond the range of values expected to have nonzero frequency in a draw of 10 × 10^100^ permutations of the 256 cell landscape lattice. This follows from the observation in [11] that a perfect checkerboard has only one microstate (if an odd number of rows or columns) or two microstates (if an even number of rows and columns) and so has the lowest possible entropy.

### 3.6. Boundary Limits of the Normal Approximation for Permuted Total Edge Length

**Figure 7:**
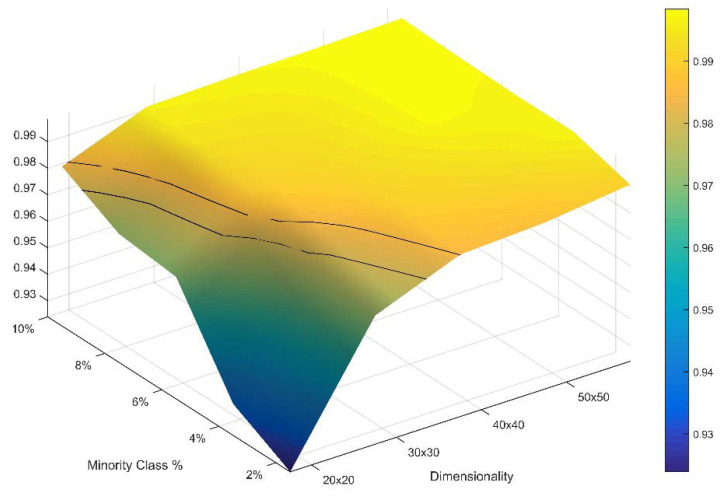
Plot of the Shapiro–Wilk *W* statistic for the distribution of total edge length for a sample of 100,000 permutations of 25 test landscapes of varying dimensionality (20 × 20, 30 × 30, 40 × 40, 50 × 50) and minority class proportion (2%, 4%, 6% 8%, 10%). The two black contours show critical values of the test statistic at alpha 0.05 for sample sizes of 1000 (lower) and 1500 (upper).

The evaluation of the normality of total edge length across a 5 × 5 factorial of landscape extent and minority class proportion of area showed that the normal assumption for the distribution of permuted total edge length is quite robust to even extreme landscape conditions in which a very small number of minority cells exist in very small landscapes (Figure S1). There were slight deviations from normality for the smallest landscapes with the largest disparity of proportionality (e.g., 20 × 20 2%, 30 × 30 2%, 20 × 20 4%, 30 × 30 4%). As expected, the departure from normality in these cases was in the form of a truncated upper end of the distribution. The departures were generally small in these cases, and in all other cases the normal approximation held. There was a non-linear relationship between the magnitude of the Shapiro–Wilk *W* statistic measuring the departure from normality and the two factor landscape extent and proportionality (Figure 7). Specifically, for landscapes above 30 × 30 pixels and with an area greater than at least 6% in the minority class, the Shaprio–Wilk *W* statistic expressed an asymptote at values above 0.99 and was not significant even with a very large sample size (1500 drawn from the permuted distribution). This shows that the normal approximation used to obtain parametric probability functions was robust and enabled the calculation of the frequency of microstates with any given total edge length macrostate in calculating the configurational entropy of landscape lattices. For all practical purposes, this demonstrates the complete robustness of the normal approximation, given that real-world analyses of landscapes virtually always deal with extents larger than 30 × 30 pixels and mosaics with more than 6% of minority class.

### 3.7. Normal Distributions of the Permuted Total Edge Length of Real Landscapes

**Figure 8:**
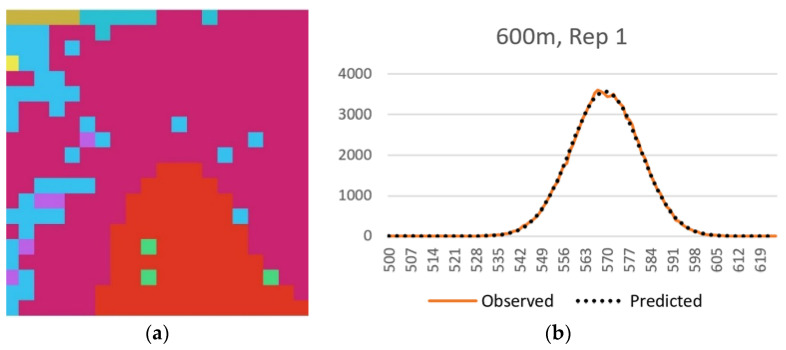
Normal distribution of the permuted total edge length for a sample landscape. (**a**) Sample landscape; (**b**) Overlay of the normal probability function predicted frequency distribution over the observed frequency distribution of total edge length resulting from 100,000 spatial randomizations of this landscape.

The 25 sample landscapes from the San Francisco Peaks region of northern Arizona varied substantially in the size (20 × 20–100 × 100 pixels), number of cover classes within them (8–52) and Shannon diversity (0.95–2.91). Despite this large difference in extent and composition, all sample landscapes produced distributions of total edge length from the permutation that were approximately normally distributed (Figure S2). Figure 8 shows this fit for one sample landscape of 20 × 20 pixel dimensionality. The observation that total edge length follows the predicted parametric normal distribution across a range of landscape extents, patch richness and evenness suggests that the approach proposed here will be robust in application to any landscape regardless of size or composition.

### 3.8. Comparison of Entropy Curves of Real Landscapes

**Figure 9:**
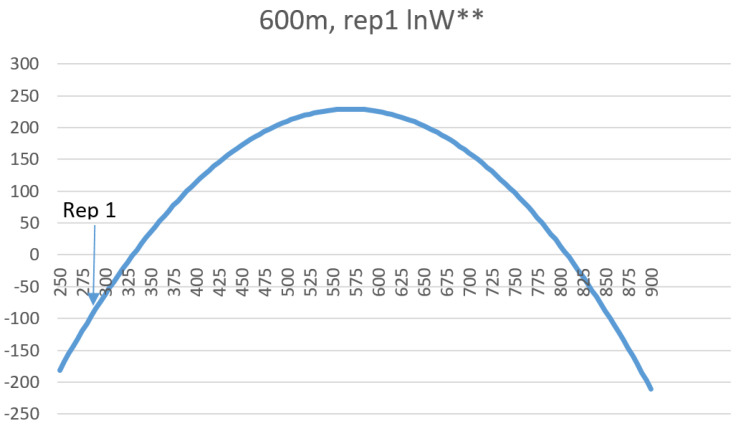
The parabolic entropy curve for a sample landscape. The actual entropy of the sample landscape is indicated with the arrow on the far left side of the curve, indicating that this landscape has very low entropy in comparison to the distribution of microstates across all possible configurational macrostates. W** is the number of microstates (configurations of the lattice) with a given macrostate (total edge length) expected in 10 × 10^100^ permutations of the lattice.

The entropy curves of the 25 sample landscapes also all perfectly followed the expected parabolic relationship (Figure S3), and the relative entropies of the sample landscapes were all very low and far to the left on the entropy curves, indicating that they are highly aggregated relative to their permuted distribution of landscape configurations and that this aggregation is a highly ordered state reflecting low configurational entropy. Figure 9 shows this fit for the same sample landscape included in Figure 8.

**Figure 10:**
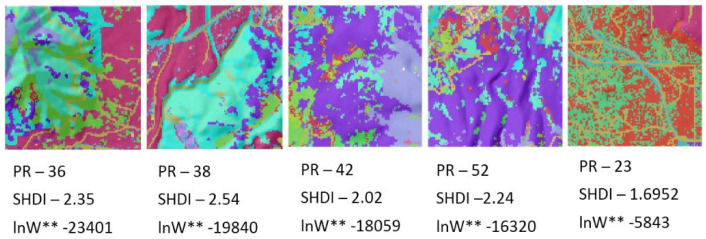
Ordering of the five replicate 100 × 100 dimensionality landscapes from low to high entropy at each of the five levels of dimensionality. SHDI is the Shannon Diversity index value for the landscape as calculated by FRAGSTATS. W** is the number of microstates (configurations of the lattice) with a given macrostate (total edge length) expected in 10 × 10^100^ permutations of the lattice.

To facilitate an intuitive understanding of the relationship between configurational entropy and landscape patterns, I ordered the five replicate landscapes with 100 × 100 dimensionality from lowest to highest entropy, for each level of dimensionality, recording the relative Boltzmann entropy for each (Figure 10). Figure 10 shows that the entropy increases with the increasing heterogeneity of the patch mosaic.

### 3.9. Predicting the Parametric Normal Distribution of Sample Landscapes

**Figure 11:**
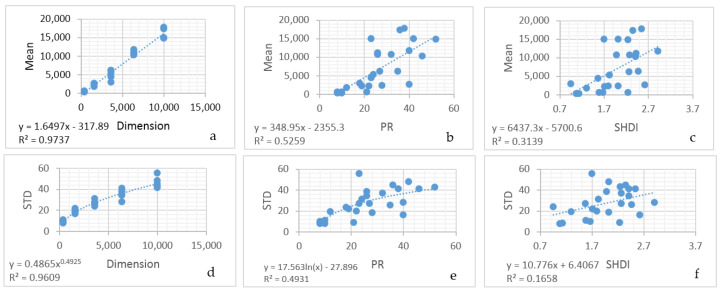
Scatter plots of the relationships between landscape dimensionality, patch richness and Shannon diversity and the mean (**a**–**c**) and standard devation (**d**–**f**) of the normal distribution of permuted total edge lengths across the 25 sample landscapes in the San Francisco Peaks region.

The entropy curves of the 25 sample landscapes were all different, in terms of their maximum, mode and the slope of their convexity (Figure S3). This follows from the fact that while the edge lengths in their permuted distributions were all normally distributed, they expressed different means and standard deviations. For configurational Boltzmann entropy to be a readily useable concept in landscape ecology it is necessary to have a functional relationship to predict the frequency distribution of permuted total edge lengths for any observed landscape. There were clear relationships between the mean and standard deviation of the permuted distribution of total edge length across the 25 sample landscapes and the dimensionality, patch richness and Shannon diversity of the sample landscape (Figure 11).

A simple linear model perfectly predicted the mean of the permuted distribution of total edge lengths as a function of landscape dimensionality, patch richness and Shannon diversity (Table 4). Specifically, the mean of the Total Edge length was a linear function of all three variables, and a three-variable linear model explained over 99% of the variance in the relationship between the mean of the total edge length in its permuted distribution across the 25 sample landscapes. Similarly, a linear model nearly perfectly predicted the standard deviation of the permuted distribution of total edge length as a function of dimensionality, patch richness and Shannon diversity (Table 5), with standard deviation of total edge length related to the square root of dimensionality, and the logarithm of patch richness, while it was linearly related to Shannon diversity.

### 3.10. Predicting the Value of Configurational Entropy Based on Landscape Extent and Pattern

I evaluated the relationship between the configurational entropy of each of the 25 sample landscapes and the extent of the landscape (dimension) and several landscape metrics, calculated by FRAGSTAT using a Generalized Linear Model (GLM) with model averaging in MuMIN library of R (R version 3.4.2 (2017-09-28), the R Foundation for Statistical Computing; Table 6). The results indicate that the dimensionality of the landscape has a strongly inverse relationship with observed configurational entropy, with landscapes with a large extent having low entropy compared to landscapes with a smaller extent. This follows from the fact that landscape patterns are highly non-random in the direction of high aggregation, and the number of permutations of a landscape pattern increases greatly with dimensionality, so that in large landscapes there will be a relatively smaller number of permuted landscape configurations with a given degree of edge length, which results in a lower metric of configurational entropy for large landscapes compared with smaller landscapes. The configurational metrics were all significantly related to configurational entropy. Specifically, configurational entropy increases with decreasing landscape heterogeneity (as measured by the aggregation index, AI), increases with increasing edge density, and decreases with increasing evenness of the landscape.

## 4. Discussion

All of the assertions that I proposed are supported by the results of this paper. First, I asserted that a distribution of microstates for any landscape mosaic can be computed by randomly permuting the lattice to produce random configurations of the same dimensionality. The results show that the vector-randomization of a matrix produces a fully randomized lattice, and repeating this a large number of times produces a distribution of randomized configurations that can be used to define the frequencies of microstates of landscape configurations. Second, I asserted that due to the central limit theorem the total edge length in the distribution of randomized landscapes would follow a normal distribution. My results show that this is the case, and that each of the 12 test landscapes produced an identical normal distribution of microstates, which indicates that the frequency of microstates in the landscape configuration is not dependent on the pattern in the focal landscape, but only on the dimensionality, number of classes, and proportion of each class in that landscape. Third, I evaluated the boundary limits of the normal approximation for small landscapes with low proportional coverage by a minority class, and found that the normal approximation is highly robust, especially for landscapes of realistic size and proportionality. Fourth, as I asserted, we can predict the proportion of microstates with any given edge length by fitting a normal probability density function to this distribution of total edge length in randomized microstates. This provides a critical parametric solution to the challenge of computing the vast number of unique configurations that can be generated from randomizing landscape lattices. Specifically, the normal probability function enables easy calculation of the expected proportion of any observed amount of total edge length in the fully permuted distribution of total edge lengths. Fifth, as I asserted, we can use the proportion of microstates (*p*), rather than the number proper (*W*), to compute the relative Boltzmann entropy, and we can use this relative entropy to compare the entropies of landscapes with the same number of pixels and cover types. Specifically, the relationship between ln*p* and ln*W* is linear with a slope of 1, allowing exact calculation of the relative differences in entropy between landscape mosaics with the same dimensionality. In addition, the relationship between configurational entropy and total edge length in a landscape of a given dimensionality, number of classes and proportion of cells in each class is parabolic, with a peak in entropy corresponding to a spatially random arrangements of cells, with entropy declining on both sides, as landscapes become more aggregated (lower total edge length) or more dispersed (higher total edge length) in pattern. This confirms my original findings for a small and simple test landscape [11].

Furthermore, by evaluating the frequency distributions of total edge length across the permutations of the configurations of 25 sample landscapes of varying size, number of cover types and evenness of proportion in each cover type I demonstrated that the parametric approximation to the normal probability density function is robust in real landscapes. This enables us to have confidence in applying the normal probability density function approach to real landscapes, and obviates the need to compute all possible configurations of a given landscape lattice in order to estimate the number of microstates in its observed macrostate of total edge length. In addition, the results show that real landscapes exhibit relative entropies that are very low, and that they depart from randomness in the direction of aggregation, as has been previously noted (e.g., [15,17] I observed that the relative entropy of the sample landscapes decreased dramatically as their dimensionality increased. This follows from the fact that (1) real landscapes are aggregated; (2) as dimensionality increases the number of possible permutations of a given landscape lattice increases greatly; and therefore (3) larger landscapes will have many more permutations that have a greater total edge length than the observed landscape. This observation also suggests that care must be taken in comparing the relative entropies of landscapes of different dimensionality, as larger landscapes will generally have lower entropy because of the effect of their size alone. Thus in order to objectively compare the relative entropies of landscapes across dimensionality it may be useful to compare the relative entropy of a given landscape to the maximum possible entropy of that landscape lattice. This can be done by dividing the observed relative entropy by the maximum value of the entropy curve for that landscape. Doing this provides a measure of the entropy of a landscape as a proportion of the maximum entropy possible for a lattice of that dimensionality, number of classes, and evenness of proportionality of amount of each cover class.

Also, by fitting linear models to predict the mean and standard deviation of the normal probability density function of the total edge length as a function of landscape dimensionality, patch richness and Shannon diversity, I demonstrated that there is a functional relationship between the entropy curve and landscape size and composition. These functions explained nearly all the variance in the parameters of the normal probability density function, which provides a critical shortcut to scientists seeking to use this method when analyzing real landscapes. Specifically, the linear equations enable a researcher to estimate the key parameters of the normal probability density function (mean and standard deviation) directly without having to permute the configuration and calculate the total edge length. Thus, with these equations researchers can directly parameterize a normal probability density function for the total edge length of any landscape, which in turn enables them to readily calculate the relative frequency of the observed total edge length and compute the lnW** value (W** is the number of microstates (configurations of the lattice) with a given macrostate (total edge length) expected in 10 × 10^100^ permutations of the lattice) of configurational entropy, as well as the index of the proportion of maximum possible entropy described above.

The model averaged GLM predictions of configurational entropy values as a function of landscape dimensionality, composition and configuration also provide important insights into how configurational entropy varies across real landscapes. First, it shows that the dimensionality of the landscape has a dominant effect of the configurational entropy, such that large landscapes tend to have smaller values of configurational entropy. This is true regardless of other factors, such as aggregation, edge density or evenness, and suggests that it is not readily possible to directly compare the configurational entropies of landscapes of different size, making configurational entropy a highly scale-dependent quantity. Scale dependence is a central focus in landscape ecology research, and future work should directly focus on exploring the scale dependence of configurational landscape entropy, by formally evaluating how configurational entropy changes with changes in the extent and grain of the landscape. Second, the model shows that configurational entropy increases with increasing landscape heterogeneity. This is expected given the fact that configurational entropy is a measure of disorder, and the most disordered state is a random configuration. Increasing heterogeneity results in the increasing disorder of the landscape mosaic, for landscapes that lie on the left side of the entropy curve (more aggregated than random), which nearly all landscapes do. Third, the model indicated that entropy decreased with increasing evenness. This at first glance is counterintuitive, given that in [11] I showed that compositional entropy increases with the increasing number and evenness of the proportional coverage of classes in a categorical map. However, this result makes sense when we understand that the configurational entropy is the logarithm of the number of ways a lattice can be arranged to produce the same total edge length, and as the evenness of the landscape increases there are many more possible permutations of the landscape (and thus higher configurational entropy), and thus fewer microstates in a given macrostate (lower configurational entropy).

I presented the basic relationship between total edge length, number of configurations with a given edge length, the Boltzmann equation, and configurational entropy [11]. However, in that paper I recognized that the formal application of the Boltzmann equation to calculate the entropy of landscape mosaics required the calculation of the total edge length of each unique configuration of the landscape, and this is intractable in landscapes of realistic size, given the vast number of possible unique spatial configurations. In that paper I proposed that one could use neutral models, such as QRULE [13], to produce large numbers of configurations that could then provide a sample distribution to compute a proportion of microstates and relative configurational entropy. The major contribution of this paper is that there is an even simpler and more powerful way to compute the configurational entropy of landscapes. Specifically, the fact that randomly permuted configurations of a landscape mosaic are normally distributed allows us to utilize the normal probability density function to calculate the expected proportion of permuted landscapes with a given edge length. Furthermore, the observation that the parameters of the normal probability density function are functions of landscape dimensionality, patch richness and diversity enables the estimation of the total edge length curve without permutations. Thus, all that is required to compute the relative Boltzmann configurational entropy of a landscape is to calculate the extent, patch richness and Shannon diversity of a given landscape and then apply the linear models presented in this paper to estimate the normal probability density function of total edge length that describes the frequency of each macrostate (different values of total edge length) possible, given the composition of that landscape. Once this normal probability density function has been defined it provides direct means to estimate the proportions of configurations (microstates) with a given total edge length (macrostate), which is the key to computing the Boltzmann entropy. In addition, the observation that there is a direct, linear relationship between ln*p* and ln*W* enables us to compute the relative configurational entropy precisely and without bias for any given landscape and quantitatively compare the entropies of different landscapes. This is the key to making the entropy concept useful in a practical way in understanding landscape patterns.

The entropy of landscape patterns is highly informative. Configurational entropy quantitatively measures the disorder of a landscape. A randomly configured landscape is the most disordered possible configuration, and our calculation of configurational entropy shows this and quantifies its degree of disorder relative to more aggregated or dispersed patterns. In addition, configurational entropy allows quantitative comparison of the degree of disorder of landscapes, which is the key foundation for using configurational entropy as a metric for quantitative landscape analysis and efforts to link pattern-process relationships to thermodynamic principles.

Quantifying the disorder of a landscape would seem to be a breakthrough that can link landscape pattern analysis to physical and ecological theory. Much of the history of landscape ecology has been devoted to the development and description of many metrics that quantify landscape patterns (e.g., [15]). However, very few landscape metrics are connected in a direct way to fundamental theory or underlying processes. The second law of thermodynamics is the fundamental theory underlying all natural change, and increasing entropy in all actions is the fundamental process that controls the emergence and decay of all structure, in landscapes or elsewhere in the universe [2,11]. Therefore, the theoretically sound and quantitative means to measure the thermodynamic disorder of landscapes developed in this paper can allow researchers to begin developing and testing theories about how pattern-process relationships operate, and to what degree they create order in landscape patterns.

In the context of research in the field of landscape ecology, how the pattern of real landscapes in natural and human modified systems fall on the distribution of configurational entropy is noteworthy (Figure 6). Real landscapes are nearly universally highly aggregated, relative to random distributions [16,17]. The test landscapes in this study were produced using a neutral model, QRULE [13], that controls the degree of aggregation, *H*. Empirical research has shown that analogs of this aggregation parameter, such as the aggregation index or contagion, in real landscapes tend to vary within a narrow range at the upper end of their theoretical distributions [16,17]. This range of high aggregation corresponds to low entropy and high order. Real landscapes are aggregated and ordered. However, the natural tendency of any natural process under the second law of thermodynamics is toward increasing disorder. The observation of a highly ordered structure at any scale requires a process that drives the creation of thermodynamic order, at the expense of the creation of more disorder in the broader system. Therefore, a promising avenue for future research would focus on quantifying the thermodynamic order of real landscapes, and then connecting those patterns to the organizing processes that create and maintain them. This provides a vehicle to transform landscape ecology from a largely descriptive field to one that explicitly links patterns and processes through the lens of the second law of thermodynamics.

### Next Steps

This paper has taken an important step forward in applying thermodynamic concepts to landscape ecology by identifying a means to efficiently calculate and compare the entropies of landscape mosaics, and clarifying the relationships between total edge length, the normal distribution, probability, microstate, macrostate, entropy and the disorder of landscape patterns. An important next step would focus on the scale-dependence of configurational entropy. The conceptualization presented here is based on a simplified model of a two-class landscape, with an equal proportion of pixels in each class, and of dimensions of 16 × 16, amounting to 256 pixels. However, a fundamental concept in geographical information systems, remote sensing and landscape ecology is that the apparent pattern of a landscape changes as one changes the resolution of analysis. Resampling a landscape mosaic to a coarser pixel resolution changes the observed pattern, as does reclassifying the number of cover types in a landscape mosaic. How this affects the entropy of a landscape mosaic is an important question, as there is no natural scale at which all landscape patterns or processes should be observed [18], and pattern-process relationships are fundamentally scale-dependent [19,20]. Understanding how the entropy of a landscape changes with variations in grain, extent and thematic resolution will be critical to integrating thermodynamic measurements and concepts into the multiscale paradigm. Gao et al. [21] have taken a first step in this regard, presenting a framework to evaluate how different levels of coarseness of resampling affects the emergent entropy of landscape patterns. Additional work is needed to clarify how entropy changes with the scale of analysis, and to link these changes to scale-dependence in pattern-process relationships.

Another avenue that promises to produce interesting work is the extent to which the landscape configurational entropy concept can be applied beyond categorical mosaics to quantify the entropy of point patterns and gradient landscapes. It has been argued that the categorical patch mosaic is an abstract simplification of what is usually a truly continuous and multi-scale, multivariate pattern in real landscapes [21,22]. To integrate the second law and the entropy concept into this multi-scale gradient paradigm we urgently need methods to robustly measure the entropy of surface patterns. Gao et al. [21] have taken an important first step in this approach, showing how one can calculate the entropy of landscape gradients using a multiscale approach in which the macrostate is a coarsened resampling of a surface, and the microstates are the individual ways that a finer grain map can be configured to produce that same macrostate. This is an important first step to integrating gradient theory and landscape entropy. A promising additional idea is analogous to the approach used here. Specifically, instead of using spatial randomization to shuffle the configuration of a categorical lattice, one could use randomization to shuffle the pixels of a continuous landscape surface. Instead of calculating the number of microstates that produce a given level of total edge length, one could calculate the number of microstates that produce the same global sum of difference between the values of a pixel and its eight neighbors. This would produce an equivalent model of the configurational entropy of a landscape gradient. Landscapes with low entropy would have low differences from their neighbors compared to the distribution of possible differences in the shuffled distribution, while landscapes with high entropy would have differences with their neighbors that are most frequent among the shuffled distribution. An analogous idea could be used in point-pattern analysis. Specifically, the long-established variance–mean ratio of a number of points within a moving box to quantify dispersion, aggregation, or randomness could be converted to a measure of entropy [11]. Specifically, point patterns producing highly aggregated or highly dispersed patterns are low in entropy, while those approaching randomness are high in entropy.

## 5. Conclusions

Why do we care? What difference does it make to be able to calculate an objective and quantitative measure of the entropy of a landscape? The most basic answer is also the most profound. All processes in nature tend to increase entropy, decrease free energy and lead to an increased dissipation of energy and increase in disorder [2]. Therefore, the observation of structure implies the action of a process that actively is organizing that structure. Ecosystems are networks of dissipative structures where the flow of energy enables the emergence of organization and structure, but at the expense of the creation of more disorder in the broader system. Landscape patterns emerge from a variety of processes acting on multiple scales. Geomorphology, including plate tectonics, orography, weathering and erosion, control major patterns of landforms and these drive many ecological processes and their effects on spatial patterns [23]. The atmosphere and ocean interact with landform and geography to drive patterns in climate gradients that control temperature and precipitation regimes, which fundamentally drive biomes [24]), species distributions [25], and community structure [26]. Human activities have fundamentally modified landscapes and ecological processes in the Anthropocene [27], and the interplay of human actions, ecological gradients, climate, and geography fundamentally drive the emergence of patterns in landscapes. I assert that we cannot understand landscapes scientifically until we can quantify their disorder rigorously, and then associate their degree of structure with processes that drive its emergence. We must link pattern and process to have a science of landscape ecology [3], and the relationships between patterns and process are fundamentally thermodynamic. Entropy and the measurement of disorder, therefore, are central to the growth of landscape ecology into a mature, theory-based, predictive science.

## Figures and Tables

**Table 1:** Investigation of the boundary limits of landscape extent and proportionality for the normal approximation. Five levels of landscape dimensionality and five levels of landscape proportion of a minority class in a two-class landscape were investigated.

Rows and Columns	Number Pixels	2% Minority	4% Minority	6% Minority	8% Minority	10% Minority
20	400	8	16	24	32	40
30	900	18	36	54	72	90
40	1600	32	64	96	128	160
50	2500	50	100	150	200	250
60	3600	72	144	216	288	360

**Table 2:** Calculating the relative Boltzmann entropy (ln*p*) for the 12 test landscapes. H1—test landscape with *H* parameter = 0.1; H2—test landscape with *H* parameter = 0.2; H3—test landscape with *H* parameter = 0.3; H4—test landscape with *H* parameter = 0.4; H5—test landscape with *H* parameter = 0.5; H6—test landscape with *H* parameter = 0.6; H7—test landscape with *H* parameter = 0.7; H8—test landscape with *H* parameter = 0.8; H9—test landscape with *H* parameter = 0.9; H10—test landscape with *H* parameter = 1.0; R—simple random test landscape; C—checker-board test landscape.

	ln*p*
H10	−167.1559064
H9	−152.5994528
H8	−147.8977559
H7	−137.2196798
H6	−122.675764
H5	−111.6424486
H4	−96.09550409
H3	−77.238565
H2	−66.53959246
H1	−47.06930617
R	−4.251511739
C	−250.09384

**Table 3:** Comparing relative configurational Boltzmann entropy (ln*p*) among the 12 test landscapes. Values are column test landscape entropy as a proportion of row test landscape entropy.

	H10	H9	H8	H7	H6	H5	H4	H3	H2	H1	R	C
**H10**	1.00	0.91	0.88	0.82	0.73	0.67	0.57	0.46	0.40	0.28	0.03	1.50
**H9**	1.10	1.00	0.97	0.90	0.80	0.73	0.63	0.51	0.44	0.31	0.03	1.64
**H8**	1.13	1.03	1.00	0.93	0.83	0.75	0.65	0.52	0.45	0.32	0.03	1.69
**H7**	1.22	1.11	1.08	1.00	0.89	0.81	0.70	0.56	0.48	0.34	0.03	1.82
**H6**	1.36	1.24	1.21	1.12	1.00	0.91	0.78	0.63	0.54	0.38	0.03	2.04
**H5**	1.50	1.37	1.32	1.23	1.10	1.00	0.86	0.69	0.60	0.42	0.04	2.24
**H4**	1.74	1.59	1.54	1.43	1.28	1.16	1.00	0.80	0.69	0.49	0.04	2.60
**H3**	2.16	1.98	1.91	1.78	1.59	1.45	1.24	1.00	0.86	0.61	0.06	3.24
**H2**	2.51	2.29	2.22	2.06	1.84	1.68	1.44	1.16	1.00	0.71	0.06	3.76
**H1**	3.55	3.24	3.14	2.92	2.61	2.37	2.04	1.64	1.41	1.00	0.09	5.31
**R**	39.32	35.89	34.79	32.28	28.85	26.26	22.60	18.17	15.65	11.07	1.00	58.82
**C**	0.67	0.61	0.59	0.55	0.49	0.45	0.38	0.31	0.27	0.19	0.02	1.00

**Table 4:** Linear model predicting the mean total edge length in the permuted distribution across the 25 sample landscapes as a function of landscape dimensionality (number of rows and columns), patch richness (number of patch types, PR) and Shannon diversity (SHDI). lm (formula = mean ~ dimension + SHDI + PR, data = data).

	Estimate	Std. Error	*t*	Pr ( > |*t*|)
(Intercept)	−3.37 × 10^3^	4.67 × 10^2^	−7.205	4.22 × 10^−7^
dimension	1.66	4.63 × 10^−2^	35.775	<2.00 × 10^−16^
SHDI	2.50 × 10^3^	3.59 × 10^−2^	6.945	7.35 × 10^−7^
PR	−7.05 × 10	1.90 × 10	−3.712	0.00129
Adjusted *R*-squared: 0.9914	*F*-statistic: 921			*p*-value: < 2.2 × 10^−16^

**Table 5:** Linear model predicting the standard deviation of total edge length in the permuted distribution across the 25 sample landscapes as a function of landscape dimensionality (number of rows and columns), patch richness (number of patch types, PR) and Shannon diversity (SHDI). lm (formula = std ~ dim^0.5^ + ln(PR) + SHDI, data = data).

	Estimate	Std. Error	*t*	Pr ( > |*t*|)
(Intercept)	4.93602	5.17294	0.954	0.351
dim^0.5^	0.48459	0.03947	12.278	4.77 × 10^−11^
ln(PR)	−0.44567	2.98704	−0.149	0.883
SHDI	−2.56384	2.33961	−1.096	0.286
Adjusted *R*-squared: 0.944	*F*-statistic: 120			*p*-value: 2.628 × 10^−13^

**Table 6:** Model-averaged coefficients from a Generalized Linear Model (GLM) model predicting configurational entropy (lnW**) as a function of landscape dimension (20 × 20, 40 × 40, 60 × 60, 80 × 80, 100 × 100) and several landscape metrics. AI—Aggregation Index, SHEI—Shannon Landscape Evenness Index, ED—Edge Density, PR—Patch Richness. W** is the number of microstates (configurations of the lattice) with a given macrostate (total edge length) expected in 10 × 10^100^ permutations of the lattice.

	Estimate	Std. Error	*z*	Pr ( > |*z*|)	AIC Variable Importance
(Intercept)	2.08 × 10^4^	1.99 × 10^4^	1.03	0.30307	
dimension	−1.90	2.05 × 10^−1^	8.758	2.00 × 10^−16^	1.0
ED	1.89	6.56 × 10^−1^	2.711	0.00672	0.49
SHEI	−3.05 × 10^4^	1.08 × 10^4^	2.668	0.00764	0.89
AI	−3.46 × 10^2^	1.24 × 10^2^	2.633	0.00846	0.40

## Supplementary Materials

The following are available online at http://www.mdpi.com/1099-4300/20/4/298/s1, Figure S1: Exploring the boundary limits of the normal approximation, Figure S2: Normal approximation for test landscapes, Figure S3: Configurational entropy curves for 15 real landscapes varying in size in the San Francisco Peaks region of Arizona.
